# CT imaging findings in symptomatic patients with and without revision surgery after reverse shoulder arthroplasty

**DOI:** 10.1007/s00256-025-04867-9

**Published:** 2025-01-18

**Authors:** Sophia S. Goller, Anna L. Falkowski, Rainer J. Egli, Georg C. Feuerriegel, Samy Bouaicha, Reto Sutter

**Affiliations:** 1 Department of Radiology, Balgrist University Hospital, Faculty of Medicine, University of Zurich, Forchstrasse 340, 8008 Zurich, Switzerland; 2 Clinic of Radiology and Nuclear Medicine, Cantonal Hospital Winterthur, Winterthur, Switzerland; 3 Department for Diagnostic, Interventional and Paediatric Radiology, Inselspital, Bern University Hospital, University of Bern, Bern, Switzerland; 4 Department of Orthopedics, Balgrist University Hospital, University of Zurich, Zurich, Switzerland

**Keywords:** Reverse shoulder arthroplasty, Revision surgery, Computed tomography

## Abstract

**Objectives:**

To evaluate CT imaging findings in symptomatic patients with and without revision surgery (RS) after reverse shoulder arthroplasty (RSA).

**Materials and methods:**

In this retrospective study, two radiologists assessed CT imaging findings in symptomatic patients with RSA over 5 years, including material fracture and loosening of the peg, baseplate, screws, and humeral stem, screw positioning, prosthesis dislocation, glenoid notching, fractures, and deltoid muscle quality. The primary outcome parameter was RS. Patients were assigned Group 1 (RS) or Group 2 (No RS).

**Results:**

Ninety-nine patients (mean age 70.4 ± 10.3 years, 61 females) met the inclusion criteria. Fifty-two patients (29 females) received RS after 34.0 ± 38.3 months. The only CT imaging finding significantly associated with RS was prosthesis dislocation (*P* = .007, odds ratio (OR) 10.95, 95% CI 1.34–89.24). All other evaluated CT imaging findings were not associated with RS. Yet, loosening of the peg (30% vs. 16%), baseplate (15% vs. 6%), and superior screw (18% vs. 7%) and periprosthetic humeral fractures (29% vs. 13%)—as common reasons for RS—were more frequent in patients with RS than in those without, however not reaching significance (*P* ≥ .11). The large majority of patients had glenoid notching (79% vs. 94%), irrespective of RS.

**Conclusion:**

In this cohort of symptomatic patients after RSA, prosthesis dislocation was the only CT imaging finding associated with RS. Besides, there was a trend with higher numbers of loosening of the peg, baseplate, and superior screw, as well as periprosthetic humeral fractures in patients with RS, though not reaching significance.

**Supplementary Information:**

The online version contains supplementary material available at 10.1007/s00256-025-04867-9.

## Introduction

Reverse shoulder arthroplasty (RSA) was first conceived by Paul Grammont in 1985 [1] and has increasingly gained popularity as a treatment for various degenerative and traumatic shoulder diseases [2]. In the beginning, the indication was limited to patients with rotator cuff (RC) arthropathy suffering from pain, loss of range of motion (ROM), and deficient activities of daily living (ADL) [3]. However, due to promising clinical outcomes, its spectrum of indications has been expanded over the last decades to conditions such as irreparable massive RC tears, displaced proximal humeral fractures, and glenohumeral osteoarthritis with significant glenoid bone deficits, even with an intact RC [4–6].

Although improvements in component designs, implant positioning, and surgical techniques have led to satisfactory long-term results [7–11], complication rates reported in the literature vary from 0 to 75%, with an overall complication rate of 9.4% and a revision rate of 2.6% after 2 years, as reported in a meta-analysis by Galvin et al. [10]. Similarly, a recent study by De La Selle et al. retrospectively evaluated a consecutive series of shoulders at a minimum follow-up of 7.4 years and indicated a revision rate of 3% [9]. Zumstein et al. evaluated events associated with RSA by distinguishing “problems” from “complications,” the latter of which has been defined as events affecting clinical outcomes [12]. While the most common problem with RSA is glenoid notching, the most common complications comprise aseptic loosening of the glenoid and humeral components, fractures, instability/dislocation, infection, and nerve injuries [12].

Due to its excellent bone contrast, CT is the first-line imaging method for preoperative implant placement planning and an indispensable component in clarifying complications after RSA [13]. Analogously, every patient in our institution receives preoperative CT imaging as a standard before revision surgery (RS) to assess the bony situation. Furthermore, CT is the imaging method to evaluate potential bony complications in patients with complaints after RSA, especially if radiographs are inconclusive. However, when assessing the need for RS after RSA, the clinical relevance of distinct imaging findings described in radiological CT reports remains to be determined.

With this background, this study aimed to evaluate CT imaging findings in symptomatic patients with and without RS after RSA.

## Materials and methods

### Patient selection

This retrospective single-center study was approved by the local institutional review board (*Cantonal Ethics Committee Zurich*) and conducted according to the principles of the Declaration of Helsinki and national ethical standards. All patients included in the data collection have given written informed consent that allows their health-related data to be used for research purposes.

**Fig. 1:**
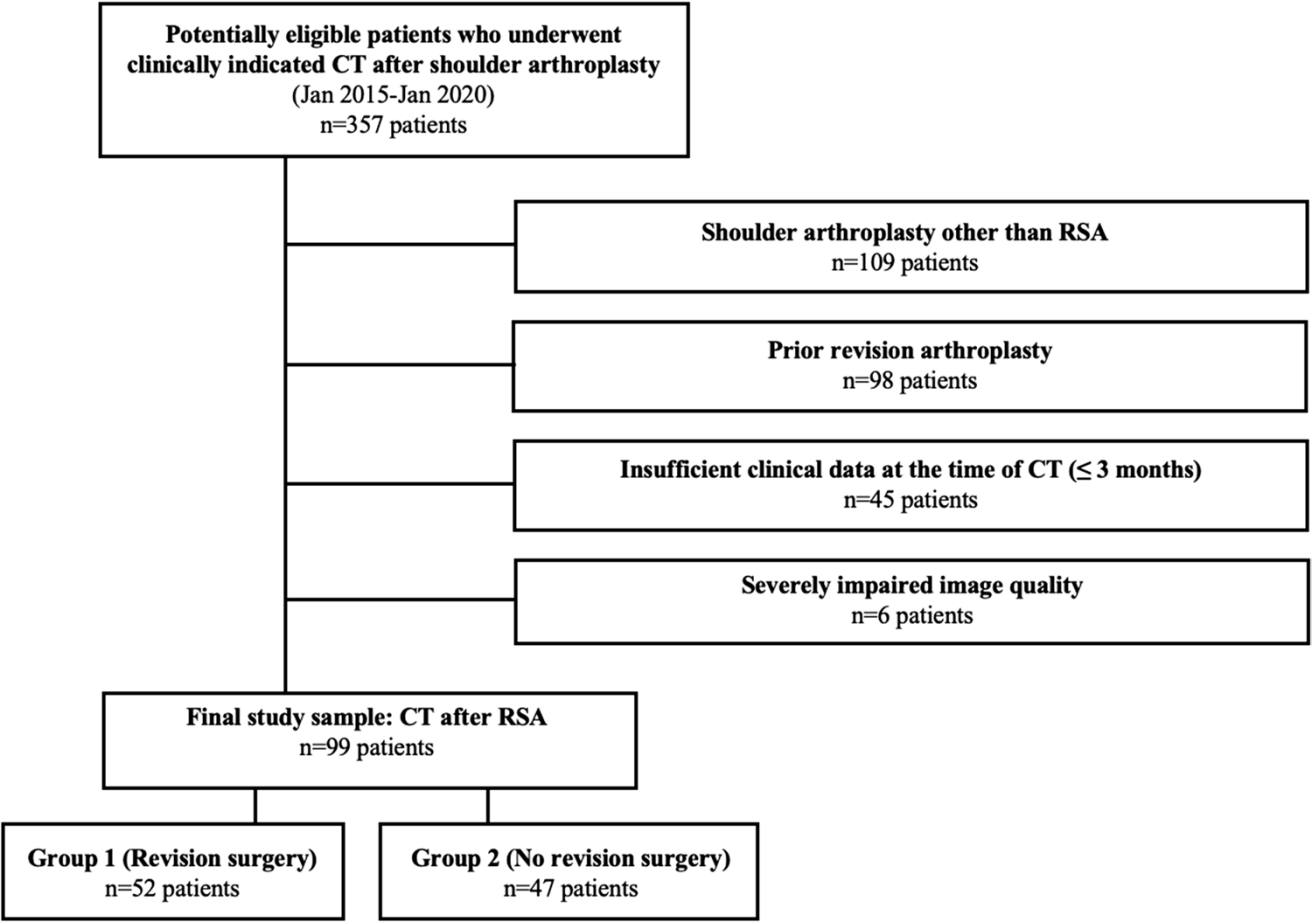
Flowchart illustrating the patient selection process. From the 357 potentially eligible patients who underwent CT after shoulder arthroplasty, 258 had to be excluded during the selection process, resulting in a final study sample of 99 patients with RSA (with one prosthesis each). Of those, 52 patients underwent revision surgery after the CT examination (Group 1), and 47 did not (Group 2). RSA, reverse shoulder arthroplasty

The institutional picture archiving and communication system (PACS) was reviewed for patients after RSA who underwent clinically indicated CT due to various complaints, including pain, restricted ROM, deficient ADL, or combinations, for further symptom clarification between January 2015 and January 2020 (*n* = 99 patients, Fig. 1). Exclusion criteria were prior revision arthroplasty, insufficient clinical data, and severely impaired image quality, e.g., due to movement. The most recent scan was evaluated if a patient received several CT examinations.

### CT imaging

Clinically indicated non-contrast standard CT of shoulders after RSA was conducted on a 128-slice CT scanner (SOMATOM Definition AS, Siemens Healthineers). For all standard CT scans, automated tube current modulation (CARE Dose4D, reference 250 mAs) was activated, and tube voltage was set to 120 kV. Further parameters were a collimation width of 0.6 mm, a rotation time of 1 s, and a pitch of 0.8. Image reconstruction in the bone kernel (Br 57) was performed with 2 mm section thickness in the axial, coronal, and sagittal planes. In addition, axial images with a 0.75 mm section thickness were reconstructed in the soft tissue kernel (Br 38). Advanced modeled iterative reconstruction (ADMIRE) strength level 3 was used for all image reconstructions. Reconstructed images in bone kernel were displayed with a window width of 2500 HU and a window level of 600 HU, while the window width and level of images in soft tissue kernel were set to 400 HU and 60 HU, respectively.

### Image analysis

**Fig. 2:**
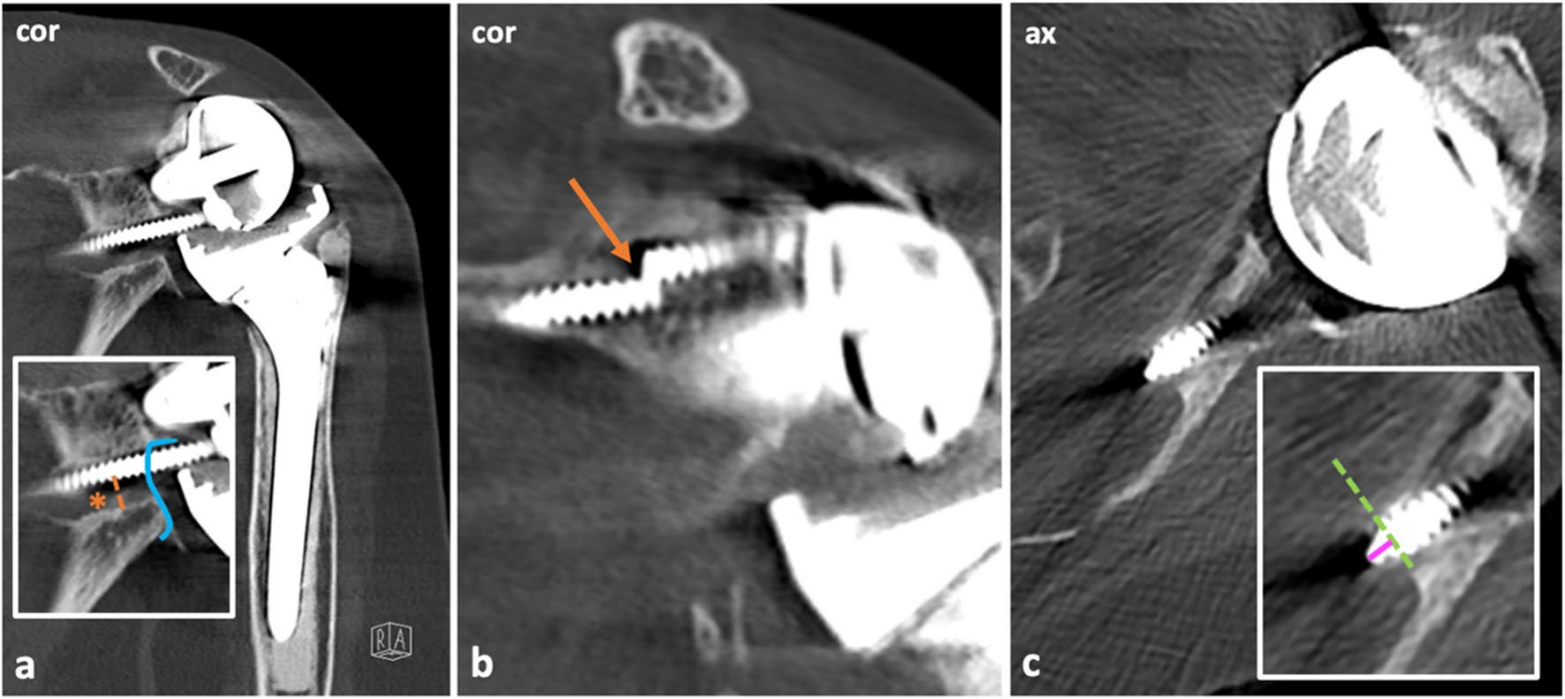
CT images illustrating the assessment of the peg and screws. Images show the left shoulder of an 87-year-old female who underwent reverse shoulder arthroplasty 4 years ago. At the time of CT imaging, the patient presented with increasing pain and restricted range of motion. There was no history of trauma. In a coronal image (**a**), severe loosening of the inferior screw was depicted (asterisk). The peg and screws were assessed regarding potential material fracture. In another coronal image (**b**), a material fracture of the superior screw was evident (arrow), which can cause instability of the prosthesis. Besides, the extent of medial protrusion of the screws due to bicortical anchoring was evaluated: This was done on axial slices **(c)** by placing a line at the bone (dashed green line) and measuring the protrusion of the screw tip perpendicular to it (pink line). Apart from this, significant glenoid notching was present (grade 3, Nerot-Sirveaux’s classification), with the notching extending over the inferior screw (blue line, **a**). Revision surgery with replacement of the glenoid component was indicated

**Fig. 3:**
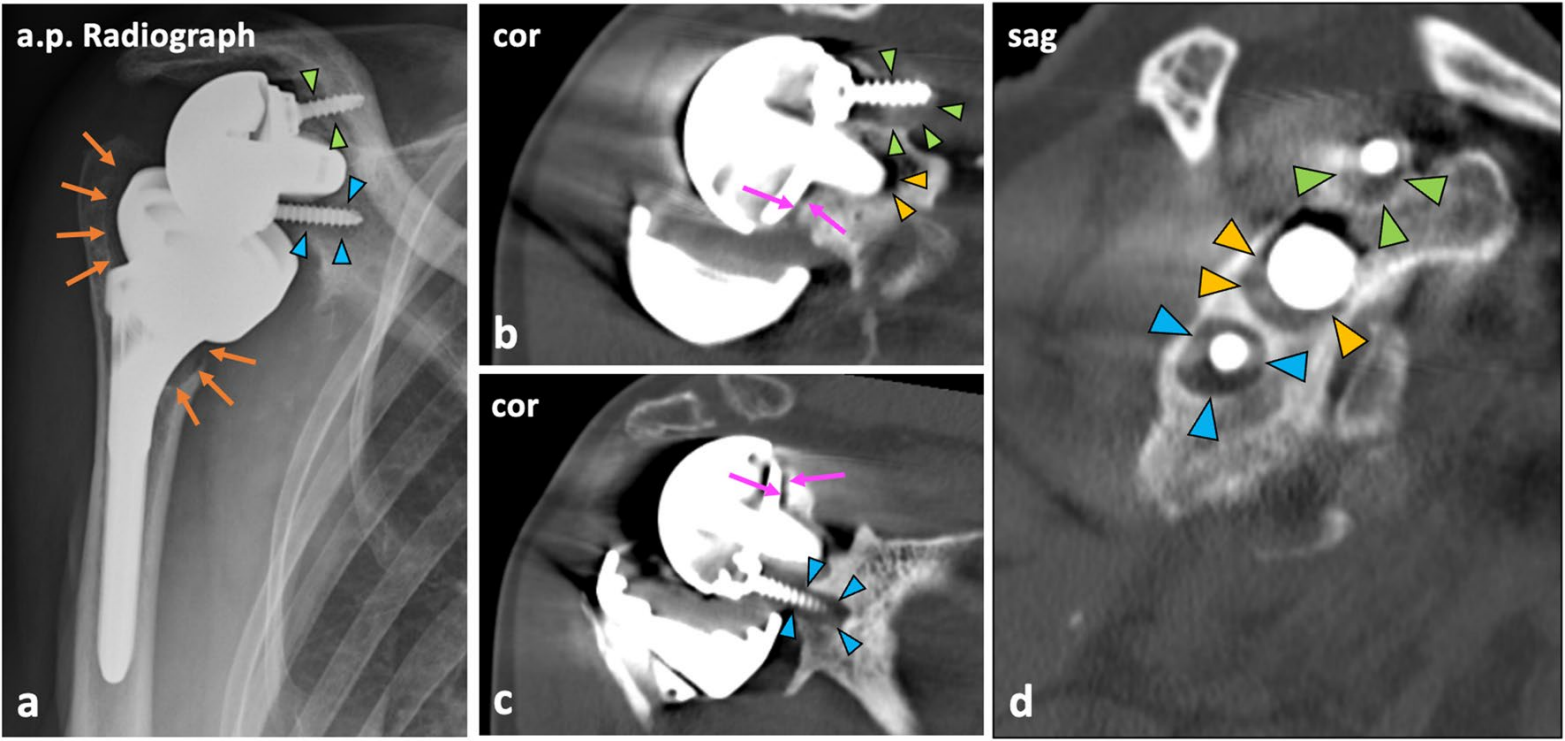
Patient example illustrating the assessment of the peg, screws, baseplate, and humeral stem. Anterior–posterior (a.p.) radiograph (**a**) and CT images (**b**–**d**) of the right shoulder of a 76-year-old female who underwent reverse shoulder arthroplasty 1 year ago and received CT imaging due to restriction of movement. The radiograph (**a**) shows significant loosening along the proximal humeral stem (orange arrows) as well as loosening of the superior (green arrowheads) and inferior screw (blue arrowheads). On coronal CT images (**b**, **c**), loosening of the screws (green and blue arrowheads) analogous to the radiograph as well as loosening of the peg (yellow arrowheads, **b**) is visible. Furthermore, the baseplate is loosened (pink arrows, **b, c**). In the sagittal image (**d**), the extent of the loosening zones around the superior screw (green arrowheads), peg (yellow arrowheads), and inferior screw (blue arrowheads) is depicted on a single slice. Besides, there was glenoid notching with touching the inferior screw (grade 2, Nerot-Sirveaux’s classification). Revision surgery with replacement of the glenoid component was indicated

**Fig. 4:**
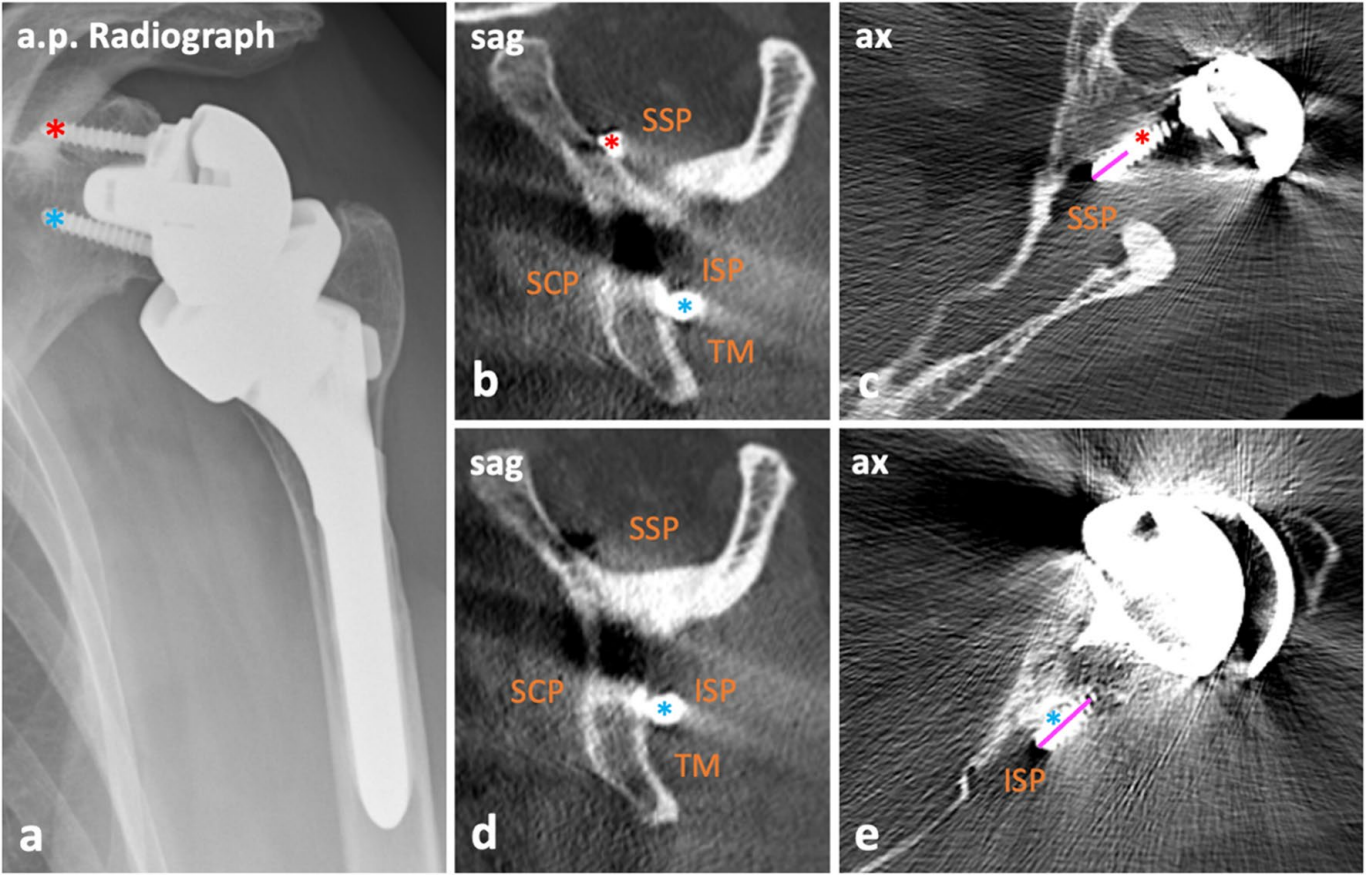
Images illustrating the assessment of medial protrusion of screw tips into the soft tissues. Anterior–posterior (a.p.) radiograph (**a**) and CT images (**b**–**e**) of the left shoulder of a 79-year-old female who underwent reverse shoulder arthroplasty 4 years ago and received CT imaging because of unclear moderate pain in the left shoulder. There was no history of trauma. Medial protrusion of the superior screw (red asterisk, **a**–**e**) into the supraspinous fossa by 4 mm (pink line, **c**) and of the inferior screw (blue asterisk, **a**–**e**) into the infraspinous fossa by 11 mm (pink line, **e**) was present. Notably, bicortical screw anchoring is intended. In this patient, an infiltration of the suprascapular nerve was recommended to narrow down the cause of pain. ISP, infraspinatus muscle; SCP, subscapularis muscle; SSP, supraspinatus muscle; TM, teres minor muscle

**Fig. 5:**
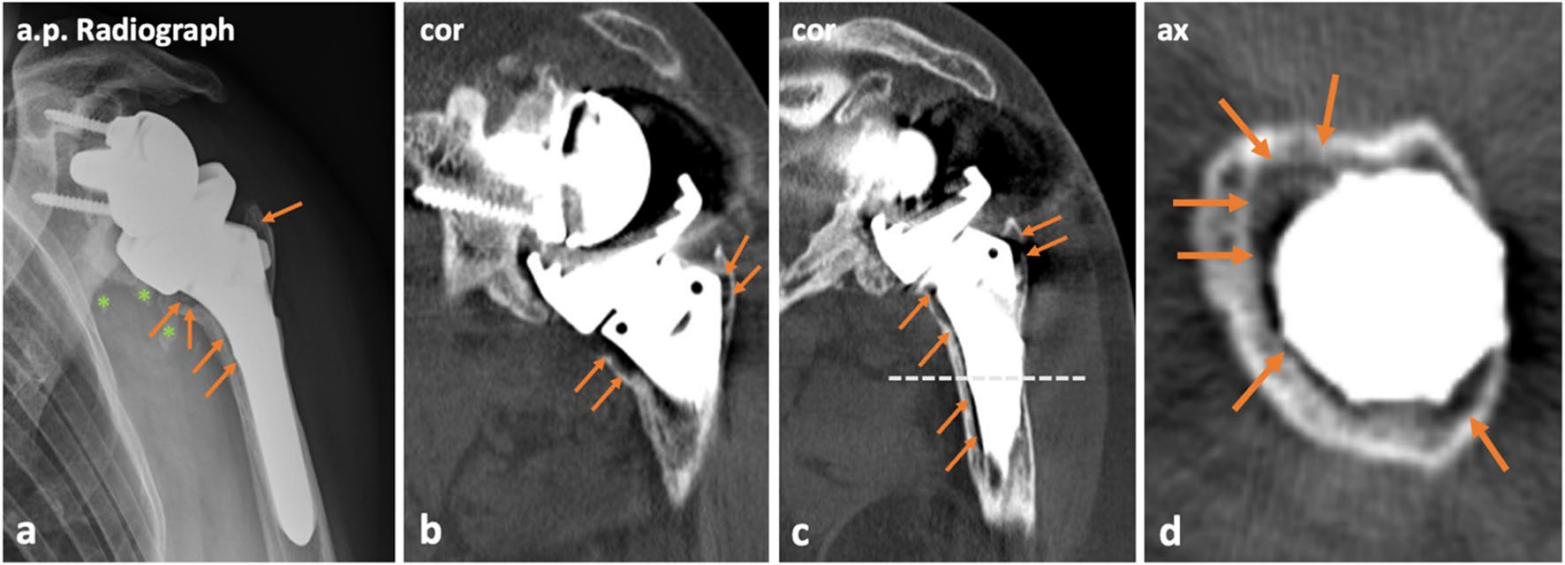
Patient example illustrating the assessment of loosening along the humeral stem. Anterior–posterior (a.p.) radiograph (**a**) and CT images (**b**–**d**) of the left shoulder of a 76-year-old female who underwent reverse shoulder arthroplasty 3 years ago. Imaging was performed due to pain after a fall on the left arm to exclude trauma sequelae. On the radiograph (**a**), loosening along the humeral stem (arrows) is already evident. Secondary findings showed some pre-existing bony fragments in projection around the glenoid component and proximal humeral stem (asterisks, **a**). On CT imaging, the loosening zones were assessable analogously to the radiograph (arrows, **b**–**d**). No revision surgery was performed in this case. The axial image (**d**) was reconstructed at the level of the middle third of the humeral stem (dashed line in **c**)

**Fig. 6:**
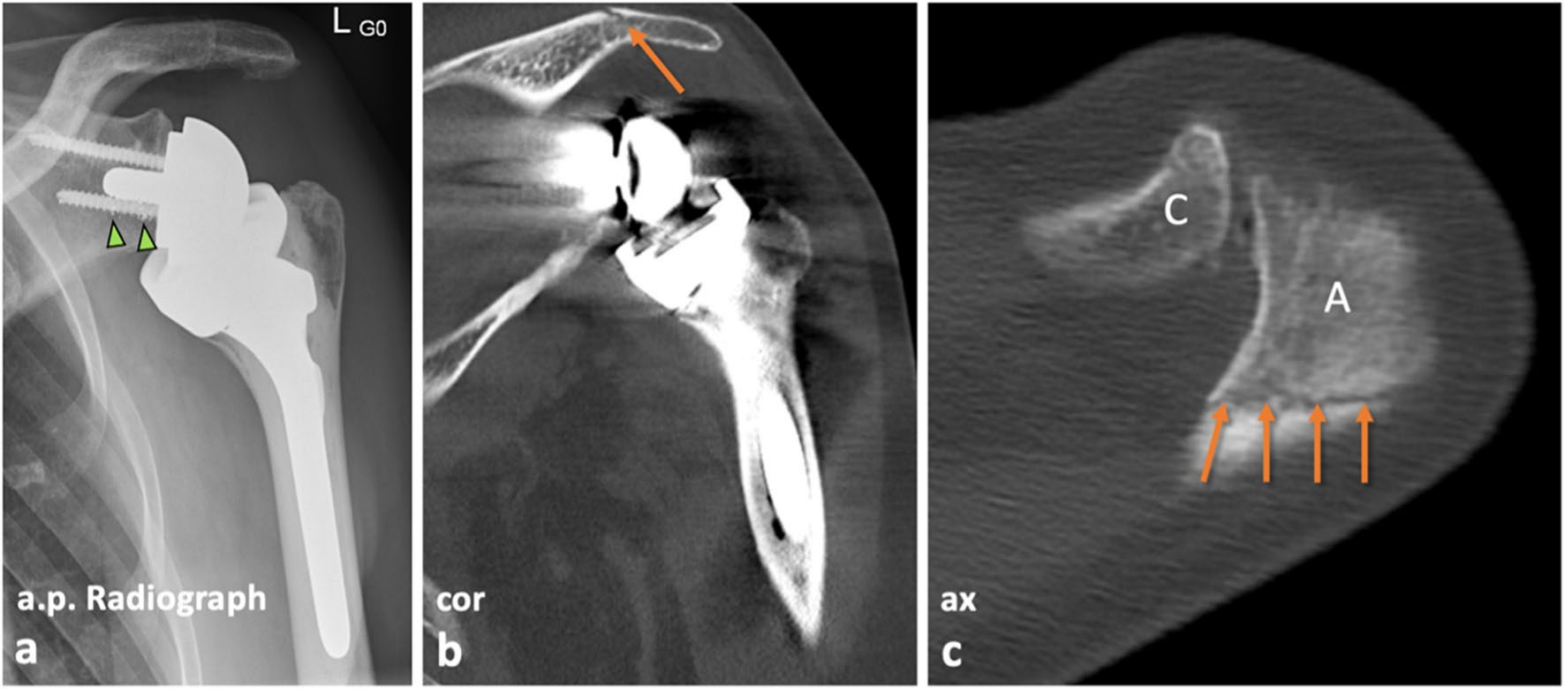
Patient example illustrating the assessment of fractures. Anterior–posterior (a.p.) radiograph (**a**) and CT images (**b**, **c**) of the left shoulder of a 71-year-old female who underwent reverse shoulder arthroplasty 2 months ago and presented with a new onset of pain. The shoulder prosthesis was intact on radiography and showed no signs of loosening. As of note, there was some metal wear around the inferior screw (arrowheads, **a**). However, CT imaging revealed the probable cause of pain, depicting a non-dislocated insufficiency fracture of the acromion (arrows **b**,** c**), which was not apparent on radiography. A conservative treatment followed. A, acromion; C, clavicle

Image analysis was done on a PACS workstation (Merlin, Phoenix-PACS). Three board-certified musculoskeletal fellowship-trained radiologists performed image analysis. All readers were blinded to clinical data and the results of the other readers. One radiologist (*SSG*, with 5 years of experience) evaluated initial postoperative radiographs for implant position. Two radiologists (*ALF* and *RJE *with 10 and 6 years of experience, respectively) independently reviewed CT studies. For each CT examination, readers had to evaluate the presence or absence of the following parameters: material fracture and loosening of the peg, baseplate, each screw (superior, anterior, inferior, and posterior), and the humeral stem. In addition, they had to determine whether there was a medial protrusion of the screw tip into the soft tissues (due to bicortical anchoring), and if so, measure its maximum extent (Figs. 2, 3, 4 and 5, Supplementary Fig. 1). Further, for each screw, readers had to assess its exact position using a clock system on sagittal slices with 12 o’clock = superior, 3 o’clock = anterior, 6 o’clock = inferior, and 9 o’clock = posterior. Also, the absence or presence of prosthesis dislocation was evaluated (Supplementary Fig. 2). Glenoid notching was assessed using Nerot-Sirveaux’s classification (grades 1–4) [14] (Figs. 2 and 3). Furthermore, the absence or presence of fractures was assessed; with this, the following types of fractures were differentiated: acromial stress fractures, coracoid fractures, scapula fractures apart from the acromion and coracoid, and periprosthetic humeral fractures (Fig. 6). Lastly, readers had to evaluate the quality of each third of the deltoid muscle (anterior, lateral, and posterior). The absence or presence of volume atrophy was assessed at the mid-glenoid level in a standardized fashion, following Meyer et al. [15]. The degree of fatty infiltration was evaluated according to the Goutallier classification (grades 0–4) [16–18]. As a supplement, each of the RC muscles (supraspinatus (SSP), infraspinatus (ISP), teres minor (TM), and subscapularis (SCP)) was assessed by determining whether there was volume atrophy using the tangent sign described by Zanetti et al. for the SSP muscle [19] and a subjective assessment (absence vs. presence) of volume atrophy for the remaining muscles. Furthermore, RC muscle fatty infiltration was graded according to the Goutallier classification on multiplanar reconstructions [16–18].

Additionally, the Constant-Murley Score (CMS) and Subjective Shoulder Value (SSV) were extracted from the clinical information system.

### Statistical analysis

All statistical analyses were performed in SPSS Statistics (v. 29, IBM Corporation). Quantile–Quantile plots and the Shapiro–Wilk test were used to test for normal distribution of continuous variables. Patients were divided into Group 1 (RS) and Group 2 (No RS). In addition to descriptive statistics, the chi-square test and Mann–Whitney *U* test were performed to assess differences between the groups. In cases of significance, odds ratio (OR) analysis was applied to quantify the strength of association between distinct CT imaging findings and RS. Percentages presented in the “Results” section were based on the results of Reader (*ALF*). To account for confounding factors, a multivariate regression model was performed to assess the influence of age, sex, time between surgery and CT scan, current or previous history of infection, and symptoms such as pain, limited ROM, and ADL deficiency on the likelihood of revision. The inter-reader agreement was analyzed by kappa statistics (Cohen’s *κ*) and intraclass correlation coefficients (ICC). The level of agreement was categorized as follows [20]: 0.0 = poor, 0.01–0.20 = slight, 0.21–0.40 = fair, 0.41–0.60 = moderate, 0.61–0.80 = substantial, 0.81–1.00 = almost perfect agreement. All statistical tests were performed two-sided, and a level of significance (*α*) of 0.05 was used.

## Results

### Patient characteristics

Table 1 summarizes patients’ demographic and clinical data in Group 1 (RS) and Group 2 (No RS). Of the 357 patients who had undergone CT after shoulder arthroplasty, 99 patients (61 females) met the inclusion criteria. The mean age of the study cohort at surgery was 70.4 ± 10.3 years, and the overall mean time interval from RSA to CT imaging was 39.8 ± 39.9 months. Each patient only had one RSA. Fifty-two patients (52.5%, 29 females) received RS after an average time of 34.0 ± 38.3 months after the initial surgery. Of the 52 patients who received RS, *n* = 22 underwent component replacement, *n* = 10 periprosthetic osteosynthesis with or without component replacement, *n* = 8 implantation of a bipolar prosthesis, *n* = 5 prosthesis explantation, *n* = 4 exchange of prosthesis, and *n* = 3 arthrolysis/debridement. The mean time interval between CT imaging and RS was 2.6 ± 6.8 months. No significant differences were observed regarding gender distribution, age, affected side, or indication for RSA between patients with RS and those without (all *P* ≥ 0.12). RC arthropathy was the most common indication for RSA in both groups (Table 1). Supplementary Table 1 shows the comparison of RC muscle quality, including volume atrophy and fatty infiltration, between Group 1 and Group 2. The mean time intervals between RSA and CT imaging differed significantly between groups: Group 1 underwent CT imaging significantly earlier after initial surgery than Group 2 (32.8 ± 40.2 vs. 47.5 ± 38.5 months, *P* = 0.003). Supplementary Table 2 presents patients’ clinical outcome parameters (CMS and SSV) up to 10 years after RSA.

**Table 1:** Demographic and clinical data in both groups of patients with reverse shoulder arthroplasty

	Group 1: RS	Group 2: No RS	*P*-value
General characteristics			
Patients	52	47	
Female	29 (55.8)	32 (68.1)	.26
Age (years)	72.2 ± 9.8	68.8 ± 10.5	.15
Right shoulder	30 (57.7)	27 (57.5)	.89
Clinical indication for RSA			
Rotator cuff arthropathy	37 (71.2)	39 (83.0)	.16
Displaced proximal humeral fracture	13 (25.0)	6 (12.8)	.12
Glenohumeral osteoarthritis*	2 (3.9)	2 (4.3)	.92
Time intervals			
RSA to CT scan (months)	32.8 ± 40.2	47.5 ± 38.5	**.003**
RSA to RS (months)	34.0 ± 38.3	N/A	
CT scan to RS (months)	2.6 ± 6.8	N/A	

The number of patients is given as a frequency with percentages in parentheses. Continuous data are presented as mean ± standard deviation. Significant results (*P* < .05) are written in bold

^*^Accompanied by glenoid bone deficits

*N/A*, not applicable; *RS*, revision surgery; *RSA*, reverse shoulder arthroplasty

### Imaging results

#### Initial postoperative radiographs

Initial postoperative radiographs were evaluated and showed a satisfactory prosthesis position in all 99 cases. There were no signs of prosthesis malposition or other complications concerning the implant.

#### CT imaging

### Peg and baseplate

None of the patients showed a material fracture of the peg. Loosening of the peg was found in more patients in Group 1 (12/52, 23.1%) than in Group 2 (7/47, 14.9%); however, this did not reach significance (*P* = 0.49). Loosening of the baseplate was seen more often in Group 1 (8/52 patients, 15.4%) than in Group 2 (3/47 patients, 6.4%); however, this did not reach significance (*P* = 0.16).

### Glenoid notching

Inferior notching of the glenoid was a prevalent finding in both groups: In Group 1, glenoid notching was present in 41/52 patients (78.9%), whereas it was seen significantly more often in Group 2: 44/47 patients (93.6%) (*P* = 0.035). Severe grade 4 glenoid notching, which reaches the central peg of the baseplate, was more frequent in patients with RS than in those without (31% vs. 17%), even though this was not statistically significant (*P* = 0.11). Table 2 shows the distribution of glenoid notching for both groups according to Nerot-Sirveaux’s classification [14].

**Table 2:** Distribution of glenoid notching grades for both groups according to Nerot-Sirveaux’s classification

	Group 1: RS	Group 2: No RS	*P*-value
Nerot-Sirveaux’s classification [14]			
Grade 1	17 (32.7)	15 (31.9)	.93
Grade 2	4 (7.7)	13 (27.7)	**.009**
Grade 3	4 (7.7)	8 (17.0)	.16
Grade 4	16 (30.8)	8 (17.0)	.11

The number of patients is given as a frequency with percentages in parentheses. Significant results (*P* < .05) are written in bold

*RS*, revision surgery

### Screws

The results regarding the position and the extent of medial protrusion of the screws are summarized in Supplementary Table 3. In summary, medial protrusion of the screw tips due to bicortical anchoring was a common imaging finding in both groups and did not show significant differences (all *P* ≥ 0.09). Its presence ranged between 44.4 and 73.1% in Group 1 and 76.6 and 100% in Group 2, depending on the respective screw and was mainly observed with the superior screw.

A material fracture of the superior screw was present in 5/52 patients (9.6%) in Group 1 and 1/47 patients (2.1%) in Group 2 (*P* = 0.12). Loosening of the superior screw was observed in 9/52 patients (17.3%) in Group 1 and 3/47 patients (6.4%) in Group 2; however, this did not reach significance (*P* = 0.10).

An anterior screw was present only in 7/52 patients in Group 1 (13.5%) and 3/47 patients (6.4%) in Group 2. There was no material fracture of any anterior screw. Loosening was exclusively seen in 1/7 patients (14.3%) in Group 1.

A material fracture of the inferior screw was present in 1/52 patients (1.9%) in Group 1 and 1/47 patients (2.1%) in Group 2 (*P* = 0.74), respectively. Loosening of the inferior screw did not differ significantly between groups (Group 1, 10/52 patients (19.2%), vs. Group 2, 8/47 patients (17.0%) (*P* = 0.39)).

A posterior screw was placed only in 9/52 patients in Group 1 (17.3%) and 3/47 patients (6.4%) in Group 2. In one single case in Group 1 (11.1%), there was a material fracture of the posterior screw. Loosening was only present in one patient in Group 1 (11.1%).

### Humeral stem

Loosening along the humeral stem was seen in 9/52 patients (17.3%) in Group 1 and 4/47 patients (8.5%) in Group 2 (*P* = 0.20).

### Prosthesis dislocation

Prosthesis dislocation was significantly more frequent in Group 1 (10/52 patients, 19.2%) than in Group 2 (1/47 patients, 2.1%) (*P* = 0.007).

### Fractures

In Group 1, 30/52 patients (57.7%) had fractures; in Group 2, fractures were seen in 19/47 patients (40.4%). Table 3 provides detailed results regarding evaluated sub-groups of fractures in both groups.

**Table 3:** Comparison of fractures between groups

	Group 1: RS	Group 2: No RS	*P*-value
Fractures			
Acromial stress fracture	8 (15.4)	7 (14.9)	.95
Coracoid fracture	4 (7.7)	3 (6.4)	.80
Other scapula fractures	3 (5.8)	3 (6.4)	.90
Periprosthetic humeral fracture	15 (28.9)	6 (12.8)	.09

The number of patients is given as a frequency with percentages in parentheses

*RS*, revision surgery; *RSA*, reverse shoulder arthroplasty

### Deltoid muscle quality

The results regarding the quality of the deltoid muscle in both groups are presented in Table 4. In Group 1 compared to Group 2, significantly more patients had volume atrophy of the lateral and posterior third of the deltoid muscle. However, the 95% CI of the OR in both cases crossed 1, implying that there is no difference between groups regarding atrophy of the lateral (*P* = 0.039, OR 4.71, 95% CI 0.96–23.05) and posterior third of the deltoid muscle (*P* = 0.029, OR 0.50, 95% CI 0.41–0.61).

**Table 4:** Comparison of deltoid muscle quality between groups

	Group 1: RS	Group 2: No RS	*P*-value
Deltoid muscle			
Anterior third			
Volume atrophy	**18 (34.6)**	**10 (21.3)**	.14
Fatty infiltration (III–IV)*	**11 (21.2)**	**6 (12.8)**	.27
0	14 (26.9)	12 (25.5)	
I	22 (42.3)	23 (48.9)	
II	5 (9.6)	6 (12.8)	
III	6 (11.5)	1 (2.1)	
IV	5 (9.6)	5 (10.6)	
Lateral third			
Volume atrophy	**9 (17.3)**	**2 (4.3)**	**.039**
Fatty infiltration (III–IV)*	**4 (7.7)**	**0 (0)**	.052
0	4 (7.7)	4 (8.5)	
I	25 (48.1)	19 (40.4)	
II	19 (36.5)	24 (51.1)	
III	3 (5.8)	0 (0)	
IV	1 (1.9)	0 (0)	
Posterior third			
Volume atrophy	**5 (9.6)**	**0 (0)**	**.029**
Fatty infiltration (III–IV)*	**3 (5.8)**	**0 (0)**	.094
0	23 (44.2)	26 (55.3)	
I	24 (46.2)	18 (38.3)	
II	2 (3.9)	3 (6.4)	
III	2 (3.9)	0 (0)	
IV	1 (1.9)	0 (0)	

The number of patients is given as a frequency with percentages in parentheses. Significant results (*P* < .05) are written in bold

^*^According to the Goutallier classification: significant fatty infiltration, comprising grades III (muscle = fat) and IV (fat > muscle) [16]

*RS*, revision surgery

### Analysis of confounding factors

To account for confounding factors, a multivariate regression model was performed to assess the influence of age, sex, time between surgery and CT scan, current or previous history of infection, and symptoms such as pain, limited ROM, and ADL deficiency on the likelihood of revision. The results showed that only moderate to severe symptoms and a current history of infection significantly impacted the outcome (*P* = 0.005 and *P* = 0.027, respectively). In addition, OR was calculated using a logistic regression model to assess the effect of different degrees of symptoms. An OR of 4.53 (95% CI 1.48–8.82, *P* = 0.008) was found for moderate to severe symptoms combined. Moderate symptoms alone showed an OR of 3.53 (95% CI 1.21–6.41, *P* = 0.02), and severe symptoms alone showed an odds ratio of 4.16 (95% CI 1.18–13.41, *P* = 0.37), which was not significant, probably due to the small number of patients.

### Inter-reader agreement

Table 5 provides the results of the inter-reader agreement analysis for individual imaging findings. In summary, the level of agreement between the two readers ranged from substantial to almost perfect.

**Table 5:** Inter-reader agreement for CT imaging findings

		Cohen’s κ*	Lower 95% CL	Upper 95% CL
Material fracture	Peg	1.000	1.000	1.000
	Superior screw	0.848	0.672	1.000
	Anterior screw	1.000	1.000	1.000
	Inferior screw	0.871	0.653	1.000
	Posterior screw	1.000	1.000	1.000
Material loosening	Peg	0.939	0.857	1.000
	Superior screw	0.726	0.565	0.887
	Anterior screw	0.711	0.327	1.000
	Inferior screw	0.852	0.734	0.969
	Posterior screw	0.714	0.334	1.000
	Baseplate	0.853	0.690	1.000
	Humeral stem	0.906	0.777	1.000
Medial protrusion	Superior screw	0.851	0.765	0.937
	Anterior screw	0.810	0.457	1.000
	Inferior screw	0.736	0.626	0.845
	Posterior screw	0.829	0.667	0.991
Screw position	Superior screw	0.812	0.667	0.957
	Anterior screw	0.815	0.470	1.000
	Inferior screw	0.820	0.695	0.945
	Posterior screw	0.846	0.558	1.000
Prosthesis dislocation		0.929	0.833	1.000
Glenoid notching		0.847	0.767	0.927
Periprosthetic glenoid fracture		0.971	0.917	1.000
Acromial stress fracture		0.922	0.814	1.000
Coracoid fracture		0.884	0.659	1.000
Other scapula fracture		1.000	1.000	1.000
Periprosthetic humeral fracture		1.000	1.000	1.000
Muscle volume atrophy	Deltoid, anterior	0.839	0.723	0.954
	Deltoid, lateral	0.912	0.790	1.000
	Deltoid, posterior	0.904	0.718	1.000
	SSP	0.861	0.763	0.959
	ISP	1.000	1.000	1.000
	SCP	0.806	0.688	0.924
	TM	0.727	0.562	0.892
Muscle fatty infiltration	Deltoid, anterior	0.874	0.796	0.952
	Deltoid, lateral	0.805	0.709	0.901
	Deltoid, posterior	0.929	0.860	0.998
	SSP	0.894	0.820	0.968
	ISP	0.877	0.801	0.953
	SCP	0.855	0.771	0.939
	TM	0.802	0.700	0.903
		ICC*		
Extent of medial protrusion	Peg	0.769	0.388	0.915
	Superior screw	0.764	0.591	0.859
	Anterior screw	0.991	0.927	0.998
	Inferior screw	0.812	0.523	0.908
	Posterior screw	0.808	0.750	0.957

^*^The level of agreement was categorized as follows [20]: .0 = poor, .01–.20 = slight, .21–.40 = fair, .41–.60 = moderate, .61–.80 = substantial, .81–1.00 = almost perfect agreement

*CL*, confidence limit; *ICC*, intraclass correlation coefficient; *ISP*, infraspinatus muscle; *SCP*, subscapularis muscle; *SSP*, supraspinatus muscle; *TM*, teres minor muscle

## Discussion

In this study, prosthesis dislocation was the only CT imaging finding associated with revision surgery (RS) after reverse shoulder arthroplasty (RSA). Besides, there was a trend with higher numbers of loosening of the peg, baseplate, and superior screw, as well as periprosthetic humeral fractures in patients with the need for RS, though not reaching significance in this study population. Glenoid notching was common in both groups (79% vs. 94%). Yet, severe grade 4 glenoid notching was more frequent in patients with RS than those without (31% vs. 17%), even though this was not statistically significant.

Our study exclusively analyzed symptomatic patients who underwent CT imaging of RSA for clinical indications (such as pain, restricted ROM, or deficient ADL), wherefore the percentage of RS in our cohort cannot be compared directly with previous clinical studies that reported reintervention rates of about 6–30% [21–24]. Yet, this can explain the observation that patients needing RS underwent CT imaging significantly earlier after initial surgery than patients without RS (32.8 ± 40.2 months vs. 47.5 ± 38.5 months), as CT imaging is part of the standard preoperative patient assessment before RS. Analogously, the time interval between CT imaging and RS was relatively short (2.6 ± 6.8 months).

The time interval between the initial RSA and RS in our study (34.0 ± 38.3 months) was similar to that reported in recent studies by Lee et al., where the reintervention occurred at 27.8 ± 23.1 months [21], and Kriechling et al., who analyzed a large cohort of 797 patients after RSA and reported a mean time interval between the initial surgery and the reintervention ranging from 14 to 21 months, depending on the specific complication [25].

Besides prosthesis dislocation, which may be accompanied by prosthesis instability, all other evaluated CT imaging findings, including peg, baseplate, screw-related parameters, glenoid notching, and deltoid muscle volume atrophy, were not associated with RS in this study cohort. Yet, regarding the loosening of the peg (30% vs. 16%), baseplate (15% vs. 6%), and superior screw (18% vs. 7%), a trend was observed for a higher presence of loosening of these prosthesis components in patients with the need for RS, compared to those without. Furthermore, there was a higher incidence of periprosthetic humeral fractures in patients with revision surgery compared to those without (29% vs. 13%). Even though these observations did not reach significance, they are essential findings when evaluating patients in potential need of RS because they represent common complications after RSA [25].

Since the glenoid screws are anchored bicortically, a certain amount of screw protrusion is intentional. However, if screws protrude too far, there may be soft tissue irritation, potentially leading to pain. Nonetheless, medial protrusion of screw tips was comparable in both groups and was not associated with RS. In this context, it is worth mentioning that anterior and posterior screws are only placed in the presence of poor bone quality, which is why screws at these positions were only present in 6–17% of patients.

Glenoid notching is mainly influenced by glenoid position and inclination and is a well-known phenomenon after RSA [26]. If notching is mild, its influence on patients’ outcomes is usually minimal [14, 27, 28]. However, it has been suggested as a cause of glenoid loosening in more severe grades [14, 29, 30]. In our study, glenoid notching was observed frequently in both groups but, interestingly, was even more common in patients who did not undergo RS than in those with RS (94% vs. 79%). The incidence of glenoid notching in our patient cohort aligns with earlier studies using conventional radiographs, reporting an incidence of glenoid notching ranging from 44 to 96% [3, 27, 29, 31]. Recently, analyzing long-term clinical and radiographic outcomes of 133 patients after RSA, Kriechling et al. reported grade 2 glenoid notching to predict an inferior outcome after RSA [24]. However, an inferior outcome does not necessarily lead to RS, wherefore, this finding cannot be compared on a one-to-one basis with our results. Still, in our cohort, grade 4 glenoid notching, which often leads to prosthesis instability, was more frequent in patients with RS than in those without (31% vs. 17%), though not reaching significance. Also of interest, severe glenoid notching may lead to material fractures of the inferior screws, which was observed approximately equally frequently in both groups at around 2%.

Another interesting finding of this study was that there was no significant difference regarding the incidence of acromial stress fractures, coracoid fractures, and other scapula fractures in patients with subsequent RS compared to those without. This might be explained by the fact that most of these fractures are treated conservatively. In our cohort, 10/52 patients who underwent RS had a periprosthetic osteosynthesis partly accompanied by component replacement. However, the incidence of fractures in our cohort of patients with CT was higher compared to previous studies; e.g., Su et al. reported an incidence of 1.4% for acromial stress fractures analyzing radiographs and clinical findings [32]. In comparison, in our study, acromial stress fractures were present in about 15% of patients in the RS cohort, as well as in patients who did not undergo RS. This difference might be explained by the projection-free fracture diagnosis on CT compared to possibly obscured fractures due to overlays on radiographs (see also Fig. 6).

Regarding deltoid muscle quality, this study found a higher volume atrophy of the lateral and posterior third of the deltoid muscle in patients who underwent RS. However, there was no significant difference regarding the degree of volume atrophy of the anterior third of the deltoid muscle, nor was there a statistically significant difference between groups concerning the extent of deltoid muscle fatty infiltration. Yet, we observed a trend for higher degrees of fatty infiltration of the deltoid muscle in patients who underwent RS. As the function of RSA depends on the deltoid muscle [33], this is an interesting aspect that, in our opinion, is worth addressing in more detail in future studies. Although clinical experience does not suggest that a deterioration in muscle quality after RSA necessarily leads to RS, it may still impair shoulder function.

Analyzing important potential confounding factors on the likelihood of RS, including age, sex, the time interval between the initial surgery and CT, current or previous history of infection, and symptoms (pain, limited ROM, deficient ADL), we found that besides a history of infection, moderate to severe symptoms significantly impacted patients’ outcome, underlining a common principle of appropriate clinical management: “Treat the patient, not the imaging finding”.

Several limitations of this study need to be addressed. First, the study design was retrospective, where clinical information was extracted from the patient charts. Second, there was a selection bias, as only symptomatic patients examined with CT at a single institution were included. Third, it must be noted that the decision for or against RS, besides imaging findings, depends on many other individual factors and possible concomitant diseases of the patients, whereby not all of them may adequately be addressed in a retrospective study setting. Despite conducting a confounder analysis for the most significant potential confounding factors, not every bias that might have influenced the surgeon’s decision for or against RS can be excluded entirely in a retrospective study design.

In conclusion, in this study, prosthesis dislocation was the only CT imaging finding associated with RS after RSA. Yet, there was a trend with higher numbers of loosening of the peg, baseplate, and superior screw, as well as periprosthetic humeral fractures in patients with the need for RS. These results emphasize the importance of radiologists and surgeons working together to find the best possible treatment option for symptomatic patients after RSA based on clinical and imaging findings.

## Supplementary Information

Below is the link to the electronic supplementary material.

Supplementary file1 (DOCX 1424 KB)

## Data Availability

This study’s data is available by the corresponding author upon reasonable request.

## Abbreviations

ADL: Activity/-ies of daily living
AUC: Area under the curve
CI: Confidence interval
CL: Confidence limit
CMS: Constant-Murley Score
HU: Hounsfield unit
ICC: Intraclass correlation coefficient
ISP: Infraspinatus (muscle)
OR: Odds ratio
PACS: Picture archiving and communication system
RC: Rotator cuff
ROC: Receiver operator characteristics
ROM: Range of motion
RS: Revision surgery
RSA: Reverse shoulder arthroplasty
SCP: Subscapularis (muscle)
SSP: Supraspinatus (muscle)
SSV: Subjective Shoulder Value
TM: Teres minor (muscle)
