# Supplementary material for: CT imaging findings in symptomatic patients with and without revision surgery after reverse shoulder arthroplasty
Source: Skeletal Radiol. 2025 Jan 18;54(8):1661–72. doi: 10.1007/s00256-025-04867-9 (PMC12174175; doi:10.1007/s00256-025-04867-9)
Supplement: Supplementary file 1 — Supplementary file1 (DOCX 1424 KB) [file 256_2025_4867_MOESM1_ESM.docx]

**Supplementary Material**

**Supplementary Table 1:** Comparison of rotator cuff muscle quality between groups.

|  | **Group 1: RS*** | **Group 2: No RS*** | *P*-value |
| --- | --- | --- | --- |
| **Rotator cuff muscles** |  |  |  |
| **SSP** |  |  |  |
| **Atrophy**** | **16 (30.8)** | **24 (51.1)** | **.040** |
| **Fatty infiltration (III-IV) ***** | **5 (9.6)** | **9 (19.2)** | .17 |
| 0 | 2 (3.9) | 3 (6.4) |  |
| I | 22 (42.3) | 10 (21.3) |  |
| II | 23 (44.2) | 25 (53.2) |  |
| III | 3 (5.8) | 6 (12.8) |  |
| IV | 2 (3.9) | 3 (6.4) |  |
| **ISP** |  |  |  |
| **Atrophy**** | **20 (38.5)** | **21 (44.7)** | .53 |
| **Fatty infiltration (III-IV) ***** | **14 (26.9)** | **14 (29.8)** | .75 |
| 0 | 1 (1.9) | 1 (2.1) |  |
| I | 20 (38.5) | 13 (27.7) |  |
| II | 17 (32.7) | 19 (40.4) |  |
| III | 5 (9.6) | 8 (17.0) |  |
| IV | 9 (17.3) | 6 (12.8) |  |
| **SCP** |  |  |  |
| **Atrophy**** | **9 (17.3)** | **6 (12.8)** | .11 |
| **Fatty infiltration (III-IV) ***** | **15 (28.9)** | **11 (23.4)** | .54 |
| 0 | 2 (3.9) | 1 (2.1) |  |
| I | 14 (26.9) | 10 (21.3) |  |
| II | 21 (40.4) | 25 (53.2) |  |
| III | 12 (23.1) | 8 (17.0) |  |
| IV | 3 (5.8) | 3 (6.4) |  |
| **TM** |  |  |  |
| **Atrophy**** | **20 (38.5)** | **11 (23.4)** | .53 |
| **Fatty infiltration (III-IV) ***** | **8 (15.4)** | **4 (8.5)** | .30 |
| 0 | 14 (26.9) | 13 (27.7) |  |
| I | 25 (48.1) | 25 (53.2) |  |
| II | 5 (9.6) | 5 (10.6) |  |
| III | 3 (5.8) | 2 (4.3) |  |
| IV | 5 (9.6) | 2 (4.3) |  |

The number of patients is given as a frequency with percentages in parentheses. Significant results (*P*< .05) are bolded.

*As of note, the majority of patients in both groups received reversed shoulder arthroplasty due to rotator cuff arthropathy (Group 1: 37/52 patients (71.2%), Group 2: 39/47 patients (83.0%).

******Atrophy of the supraspinatus muscle has been determined using the tangent sign by Zanetti [19], while the infraspinatus, subscapularis, and teres minor muscles have been subjectively assessed (absence vs. presence of volume atrophy).

***********According to the Goutallier classification: Significant fatty infiltration, comprising grades III (muscle=fat) and IV (fat >muscle) [16].

*ISP, infraspinatus muscle; RS, revision surgery; SCP, subscapularis muscle; SSP, supraspinatus muscle; TM, teres minor muscle.*

**Supplementary Table 2:** Comparison of clinical outcome parameters between groups.

|  | **Group 1: RS** | **Group 2: No RS** | *P*-value |
| --- | --- | --- | --- |
| **Clinical outcome parameters** |  |  |  |
| **0 y *** |  |  |  |
| CMS | 31.1 ± 13.8 | 30.9 ± 16.4 | .68 |
| SSV | 33.0 ± 20.8 | 29.0 ± 19.3 | .44 |
| **2 y**** | 31.0 ± 24.2****** | 32.8 ± 13.9****** | .06 |
| CMS | 38.6 ± 21.7 | 45.5 ± 18.0 | .08 |
| SSV | 41.3 ± 25.3 | 54.4 ± 24.6 | **.016** |
| **5 y**** | 51.9 ± 30.0****** | 51.8 ± 16.7****** | .28 |
| CMS | 38.1 ± 21.9 | 48.0 ± 18.2 | **.025** |
| SSV | 45.6 ± 29.3 | 54.5 ± 24.5 | .13 |
| **7 y**** | 65.9 ± 25.3****** | 77.4 ± 19.9****** | **.016** |
| CMS | 37.5 ± 22.3 | 50.0 ± 20.3 | **.035** |
| SSV | 39.4 ± 30.1 | 60.8 ± 29.2 | **.010** |
| **10 y**** | 119.7 ± 21.2****** | 158.1 ± 37.3****** | .06 |
| CMS | 44.8 ± 26.7 | 52.0 ± 22.5 | .54 |
| SSV | 53.1 ± 32.2 | 61.6 ± 31.7 | .52 |

Continuous data are presented as mean ± standard deviation. Significant results (*P*<.05) are bolded.

* Preoperative assessment data (collected the day before reverse shoulder arthroplasty).

** Postoperative clinical outcome parameters were assessed approximately 2, 5, 7, and 10 years after RSA; exact time intervals in months are indicated above clinical outcome parameters for each time point.

*CMS, Constant-Murley Score; RS, revision surgery; SSV, Subjective Shoulder Value; y, years (after RSA).*

**Supplementary Table 3:** Position and mean diameter of medial protrusion of screws in both groups.

|  | **Group 1: RS** | **Group 2: No RS** | *P*-value |
| --- | --- | --- | --- |
| **Position*** |  |  |  |
| **Superior screw** |  |  |  |
| 11 | 0 (0) | 2 (4.3) |  |
| 12 | 38 (73.1) | 43 (91.5) |  |
| 1 | 13 (25.0) | 2 (4.3) |  |
| 2 | 1 (1.9) | 0 (0) |  |
| **Anterior screw** |  |  |  |
| 2 | 1 (14.3) | 1 (33.3) |  |
| 3 | 5 (71.4) | 1 (33.3) |  |
| 4 | 1 (14.3) | 0 (0) |  |
| 5 | 0 (0) | 1 (33.3) |  |
| **Inferior screw** |  |  |  |
| 5 | 2 (3.9) | 3 (6.1) |  |
| 6 | 38 (73.1) | 41 (83.7) |  |
| 7 | 11 (21.2) | 3 (6.1) |  |
| 8 | 1 (1.9) | 0 (0) |  |
| **Posterior screw** |  |  |  |
| 8 | 1 (11.1) | 1 (33.3) |  |
| 9 | 6 (66.7) | 1 (33.3) |  |
| 10 | 2 (22.2) | 1 (33.3) |  |
| **Medial protrusion [mm]**** |  |  |  |
| **Superior screw** | 5.2 ± 3.4 | 5.7 ± 3.1 | .39 |
| **Anterior screw** | 5.0 ± 6.3 | 9.0 ± 10.6 | .48 |
| **Inferior screw** | 6.2 ± 3.4 | 5.4 ± 3.4 | .24 |
| **Posterior screw** | 8.3 ± 4.3 | 6.0 ± 3.4 | .29 |

The number of patients is given as a frequency with percentages in parentheses. Continuous data are presented as mean ± standard deviation.

* Screw positions were assessed using a clock system on sagittal slices with 12 o’clock=superior, 3 o’clock=anterior, 6 o’clock=inferior, and 9 o’clock=posterior.

** Due to intended bicortical anchoring of screws.

*RS, revision surgery.*


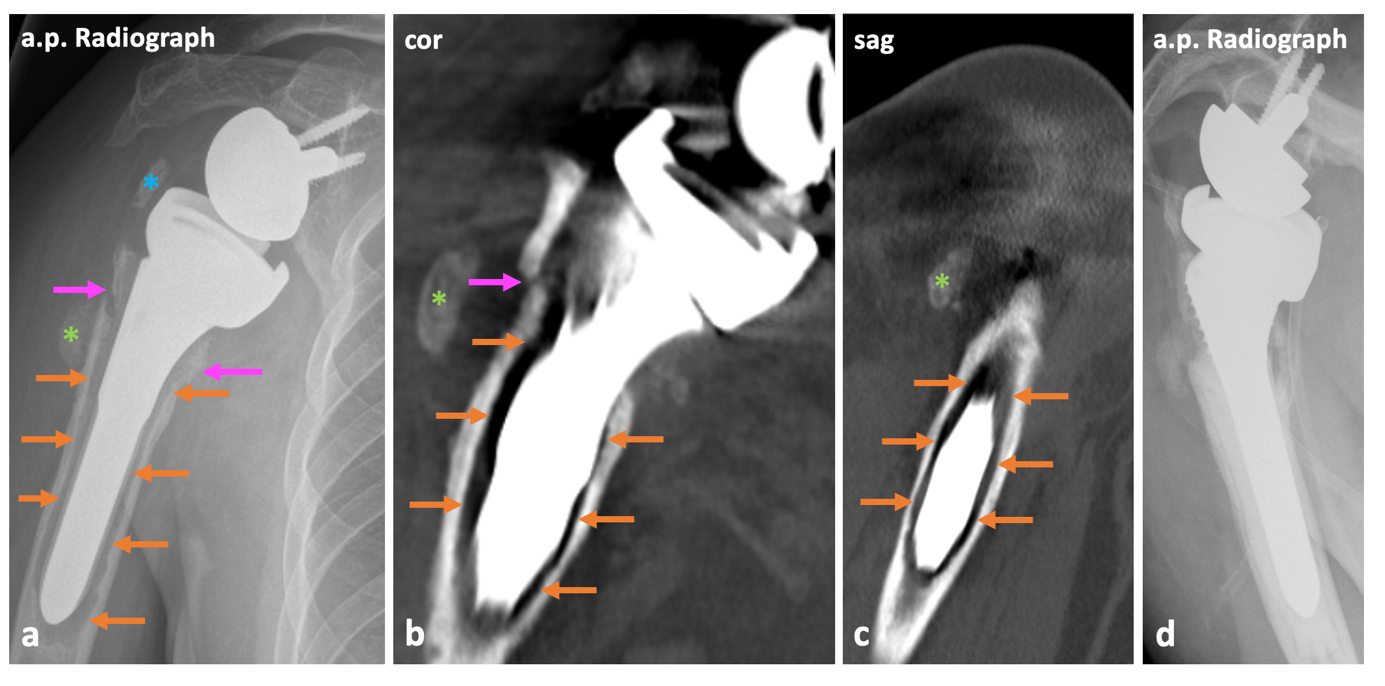


**Supplementary Figure 1:** Patient example illustrating severe loosening along the humeral stem and subsequent revision surgery due to a periprosthetic humeral shaft fracture.

Anterior-posterior (a.p.) radiograph **(a)** and CT images **(b, c)** of the right shoulder of a 47-year-old female who underwent reverse shoulder arthroplasty two years ago after a displaced proximal humeral fracture. The patient had a known peri-prosthetic proximal humeral shaft fracture for several months, and CT imaging **(b, c)** was performed for planning revision surgery. The known peri-prosthetic proximal humeral shaft fracture was visualized on radiography (pink arrows, **a**). Furthermore, there was severe loosening along the humeral stem (orange arrows, **a**), which was also depicted on the CT images (orange arrows, **b, c**). The greater tubercle (blue asterisk, **a**) was proximally displaced. The green asterisk **(a-c)** marks a fracture-associated ossification/ callus formation. Revision surgery with replacement of the humeral stem was performed: The radiograph after revision surgery **(d)** revealed a regular postoperative outcome after cemented stem placement.


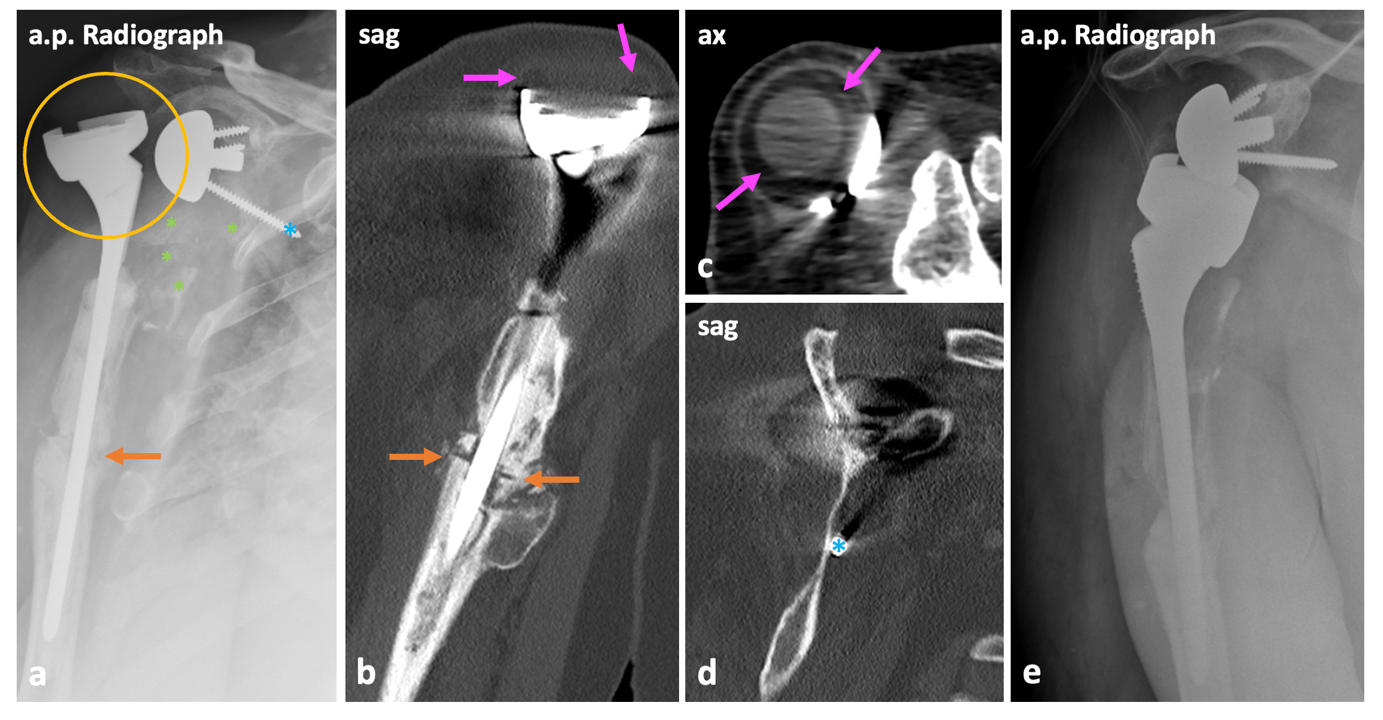


**Supplementary Figure 2:** Patient example illustrating prosthesis dislocation with subsequent revision surgery.

Anterior-posterior (a.p.) radiographs **(a, e)** and CT images **(b-d)** of the right shoulder of a 66-year-old male who underwent reverse shoulder arthroplasty 14 years ago. Radiography **(a)** and CT imaging **(b-d)** were performed due to suspected atraumatic prosthesis dislocation. On radiography **(a)**, superolateral dislocation with increased external rotation of the humeral component was diagnosed (circle). Besides, there was a non-union of the humeral shaft with associated hypertrophic callus formation (orange arrows, **a, b**) as well as multiple peri-articular osseous fragments (green asterisks, **a**), which were a sequelae of a periprosthetic shaft fracture two years ago. The polyethylene inlay was still on the humeral shaft component (pink arrows, **b, c**). Furthermore, there was a medial protrusion of the inferior screw (blue asterisk, **a, d**) by 10 mm into the subscapular fossa. This patient underwent revision surgery with replacement of the humeral stem. The postoperative radiograph **(e)** showed correct articulation of the components.
